# Single Molecule Real-Time Sequencing and Traditional Cultivation Techniques Reveal Complex Community Structures and Regional Variations of Psychrotrophic Bacteria in Raw Milk

**DOI:** 10.3389/fmicb.2022.853263

**Published:** 2022-02-10

**Authors:** Bingyao Du, Lu Meng, Huimin Liu, Nan Zheng, Yangdong Zhang, Shengguo Zhao, Jiaqi Wang

**Affiliations:** ^1^State Key Laboratory of Grassland Agro-Ecosystems, Key Laboratory of Grassland Livestock Industry Innovation, Ministry of Agriculture and Rural Affairs, College of Pastoral Agriculture Science and Technology, Lanzhou University, Lanzhou, China; ^2^Key Laboratory of Quality & Safety Control for Milk and Dairy Products of Ministry of Agriculture and Rural Affairs, Institute of Animal Sciences, Chinese Academy of Agricultural Sciences, Beijing, China; ^3^Laboratory of Quality and Safety Risk Assessment for Dairy Products of Ministry of Agriculture and Rural Affairs, Institute of Animal Sciences, Chinese Academy of Agricultural Sciences, Beijing, China

**Keywords:** milk, PacBio single molecule real-time sequencing, psychrophilic bacteria, community structures, regional

## Abstract

In this study, we investigated the species composition and diversity of psychrotrophic bacteria in raw milk from Heilongjiang, Inner Mongolia, Gansu, Henan, Anhui, Jiangsu, Chongqing, and Hunan provinces in China using traditional cultivation and PacBio Single Molecule Real-Time sequencing methods. The isolated psychrotrophic bacteria were highly diverse, which composed of 21 genera and 59 species. *Pseudomonas* accounted for 58.9% of the total genera while *Stenotrophomonas* and *Enterococcus* were also highly represented (above 5.0%). In particular, *P. azotoformans* occurred at a level of 16.9% and *P. paralactis, P. lactis, E. faecalis, and P. marginalis* were present in relatively high proportions (above 4.0%). Regional differences were found significantly among the test regions except samples from Heilongjiang and Inner Mongolia were similar. Additionally, differences were observed between days in Henan, Anhui, and Jiangsu samples. Therefore, control strategies must be implemented on regional and season basis.

## Introduction

Milk is an indispensable food for humans and its high nutrient content plays a key role in maintaining health especially for the elderly and children. The quality and safety of dairy products is deeply trusted by consumers (America Department of Health and Human Services, 2019) and the high level of safety procedures in the dairy industry have all but eliminated any physical and chemical dangers of milk consumption (Wang et al., 2018; Du et al., 2019). In contrast, milk contamination by microorganisms is still a constant threat and raw milk may be exposed to microorganisms during milking, storage, transportation, and processing. This risk is offset by the use of rapid cooling equipment and cold chain systems in the dairy industry. The temperature of the raw milk after extrusion is rapidly reduced to <6°C and kept at this level during storage and transportation. Interestingly, the presence of psychrotrophic bacteria can result in contamination of the milk even at these storage temperatures (Ahmed et al., 2014; Fusco and Quero, 2014; Hahne et al., 2019; Du et al., 2020).

Psychrophilic bacteria are widely distributed in the environment and can contaminate cow udders, milking equipment and storage tanks. Cold storage conditions provide ideal conditions for their growth (Yuan et al., 2019). These organisms also secrete heat-resistant proteases and lipases that can significantly alter the quality of dairy products. For example, lipid hydrolysis by contaminating lipases can lead to rancidity while released proteases hydrolyze casein and produces peculiar smells and cause aging and gelation thereby shortening the shelf life (Xin et al., 2017; Meng et al., 2018; Yang et al., 2020; Bu et al., 2022).

The primary species of psychrotrophic bacteria in raw milk have been identified in the genera *Pseudomonas, Chromobacterium, Clostridium, Lactobacillus, Alcaligenes, Flarobacterium, Micrococcus, Corynebacterium, Streptococcus*, and *Enterobacterium* (Wang et al., 2018). This diversity was further cataloged in raw milk samples from Heilongjiang Province in Northern China and psychrophilic milk bacteria were primarily found for the genera *Chryseobacterium, Pseudomonas, Staphylococcus, Acinetobacter, Clostridium, Flavobacterium, Lactococcus, Kocuria, Bacillus, Serratia, Sphingobacterium*, and *Aerococcus* (Yang et al., 2020). However, the diversity of psychrotrophic bacteria in raw milk for different regions of China was not clear. Therefore, this study was focused on the identification of psychrophilic bacterial community structures in raw milk from different regions of China through a combination of traditional culture and PacBio single-molecule real-time sequencing. We also examined whether regional differences in the community structure of psychrophiles were present. These results provide suggestions for the prevention and control of psychrotrophic bacteria in raw milk.

## Materials and Methods

### Sampling

From December 2018 to 2020, eight farms were selected from eight regions in China for raw milk collection. The regions included Hunan, Henan, Heilongjiang, Chongqing, Inner Mongolia, Gansu, Anhui, and Jiangsu. At each ranch, one batch of samples was collected every day for 3 consecutive days except for Henan when sample collection was 4 consecutive days. The milk was immediately placed in 200 mL sterile bottles and refrigeration at 4°C for pending analysis.

### Psychrophilic Bacterial Culture

Psychrophiles were cultured using 25 mL raw milk that was diluted 10 times and 200 μL of three suitable dilutions were spread on Psychrophilic bacterial selective agar plates (Qingdao Hope Bio-Technology Co., Ltd., Qingdao, China). The plates were incubated at 6.5°C incubator for 10 d according to IDF method (IDF 101:2005) (International Dairy Federation, 2005). Single colonies were then streak-purified until pure cultures were obtained. Randomly selected colonies were used for identification in triplicate.

### Sequence Analysis of Bacterial Colonies

DNA was extracted from individual bacterial colonies on agar plates and directly lysed in PCR tubes containing 50 μL lysis buffer (Takara Biomedical Technology, Beijing, China) that was heated at 80°C for 15 min. Bacterial species were identified by PCR using 16S rRNA universal primers (Table 1). The PCR reactions were carried out using Emerald Amp Max PCR Master Mix (Takara Biomedical Technology, Beijing, China) using 2 μL DNA template and other conditions as specified by the manufacturer. The PCR amplification program for 16S rRNA was as follows: 94°C 4 min, 30 cycles of 94°C for 30 s, 57°C for 30 s and 72°C for 90 s and a final step of 72°C for 10 min.

**TABLE 1:** Primer sequences used in the study.

Gene	Primers (5′ to 3′)	Reference
16S rRNA	AGAGTTTGATCCTGGCTCAG	Scarpellini et al., 2004
	CTACGGCTACCTTGTTACGA	

Amplicons were electrophoresed through 1.5% agarose gels to determine whether a target band was present. The PCR products of 16S rRNA amplification were purified and sequenced by a commercial company [BGI Tech Solutions (Beijing Liuhe), Beijing, China].

### Direct Sequence Analysis of Milk Samples

Milk samples (10 mL each) were centrifuged at 14,000 × *g* for 5 min and the precipitate was used for DNA extraction using the PowerSoil DNA Isolation kit (Mobio, Carlsbad, CA, United States) according to the manufacturer’s instructions. Extracted DNA was amplified using the following PCR cycling program: 95°C 5 min, followed by 30 cycles of 95°C 30 s, 50°C 30 s, 72°C 90 s and a final extension at 72°C for 7 min. The PCR primers (5′- 3′) specific for the 16S rDNA were 27F (GAGAGTTTGATCCTGGCTCAG) and 1492R (AAGGAGGTGATCCAGCCGCA) (Wang et al., 2018). The amplified products were purified using MagicPure Size Selection DNA Beads (TransGen Biotech, Beijing, China). Electrophoresis results were quantified using Image J software^1^ and a Sequel II kit (PacBio, Menlo Park, CA, United States) was used for sequence analysis following the instructions of the manufacturer.

### Statistical Analyses

The raw data reads were corrected to obtain the CCS (Circular Consensus Sequencing) sequence using SMRT Link (version 8.0) and lima (v1.7.0) software to identify the CCS sequence through the barcode sequence. Chimeras were removed using UCHIME version 8.1 to obtain the final high-quality CCS sequences (Edgar et al., 2011). The composition of psychrotrophic bacterial microbiota was investigated by submitting the psychrotrophic bacteria database to the raw milk microbiota database. Using the sequence similarity relationship between valid data, different data clusters were grouped in operational taxonomic units (OTU) at a 97% level and annotated based on OTU abundance and sequence information (Dixon, 2003). The relative abundance map of microbial community composition was carried out in R ggplot2 (version 2.2.1) (Wickham, 2016), the diversity index was calculated using https://view.qiime2.org, and the principal coordinate analysis (PCoA) was generated in the R project Vegan package version 2.5.3 (Dixon, 2003), Heat map, Venny, and linear discriminant analysis were generated using the online software at https://www.genescloud.cn.

## Results

### Identification of Cultivable Psychrophilic Bacteria in the Sample

In the current study we examined 248 different psychrophilic bacterial colonies that were isolated from 25 raw milk samples, which identified as 59 species in 21 genera. The dominant genus was *Pseudomonas* (58.9%) with *Stenotrophomonas* (7.3%), *Enterococcus* (6.5%), *Chryseobacterium* (4.8%), and *Serratia* (3.6%) as secondary dominants. *P. azotoformans* accounted for 16.9% of the total species followed by *P. paralactis* (7.7%), *P. lactis* (5.2%), *E. faecalis* (4.8%), *and P. marginalis* (4.0%) (Table 2).

**TABLE 2:** Identification and distribution of psychrotrophic bacteria isolated from raw milk samples collected from different regions in China.

Genus (Total No.)	Species (No.)	Genus (Total No.)	Species (No.)
*Pseudomonas*(146)	*Azotoformans* (42)	*Chryseobacterium* (12)	*hominis*(8)
	*Paralactis* (19)		*lactis*(2)
	*lactis*(13)		*cucumeris*(1)
	*marginalis*(10)		*haifense*(1)
	*helmanticensis*(9)	*Serratia*(9)	*marcescens*(7)
	*hibiscicola*(8)		*surfactantfaciens*(2)
	*gessardii*(7)	*Bacillus*(8)	*galactosidilyticus*(6)
	*oryzihabitans*(5)		*licheniformis*(1)
	*mucidolens*(4)		*kochii*(1)
	*lundensis*(3)	*Moraxella*(8)	*osloensis*(8)
	*fluorescens*(3)	*Lactococcus*(5)	*raffinolactis*(2)
	*reinekei*(3)		*lactis*(2)
	*koreensis*(3)		*garvieae*(1)
	*canadensis*(3)	*Rothia*(5)	*endophytica*(5)
	*lurida*(3)	*Comamonas*(3)	*testosteroni*(3)
	*weihenstephanensis*(2)	*Citrobacter*(3)	*freundii*(3)
	*rhodesiae*(1)	*Staphylococcus*(3)	*xylosus*(2)
	*synxantha*(1)		*gallinarum*(1)
	*kilonensis*(1)	*Macrococcus*(3)	*goetzii*(2)
	*coleopterorum*(1)		*caseolyticus*(1)
	*poae*(1)	*Acinetobacter*(2)	*baumannii*(2)
	*fildesensis*(1)	*Enterobacter*(1)	*bugandensis*(1)
	*tolaasii*(1)	*Psychrobacter*(1)	*faecalis*(1)
	*baetica*(1)	*Sporosarcina*(1)	*luteola*(1)
	*orientalis*(1)	*Streptococcus*(1)	*suis*(1)
*Stenotrophomonas*(18)	*maltophilia*(9)	*Kocuria* (1)	*arsenatis*(1)
	*pavanii*(6)	*Galactobacter*(1)	*valiniphilus*(1)
	*rhizophila*(3)	*Pantoea*(1)	*agglomerans*(1)
*Enterococcus*(16)	*faecalis*(12)		
	*pseudoavium*(3)		
	*hirae*(1)		

### Community Structure Analysis

**FIGURE 1:**
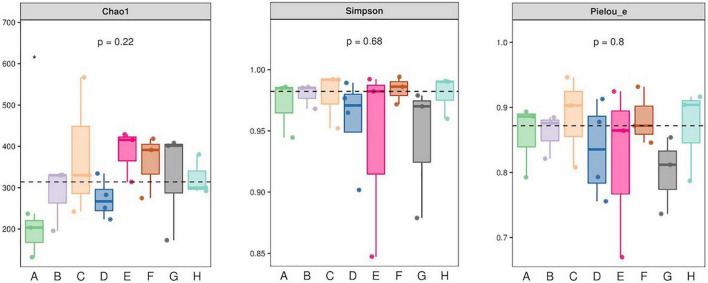
Alpha analysis metrics for microbiota present in milk samples collected in eight locations for this study. A. Heilongjiang, B. Inner Mongolia, C. Gansu, D. Henan, E. Anhui, F. Jiangsu, G. Chongqing, and H. Hunan. *Significant difference.

Single-molecule real-time sequencing was used to determine the identities of microorganisms in the raw milk samples. The richness, uniformity and coverage of the sample sequencing were sufficient to predict the diversity within each sample. Interestingly, the samples from Heilongjiang possessed significantly lower Chao 1 species richness scores than samples from the other regions. However, the Simpson and Pielou evenness indices were not significantly different (Figure 1). These data indicated that the microbial diversity between the sampling locations was similar.

**FIGURE 2:**
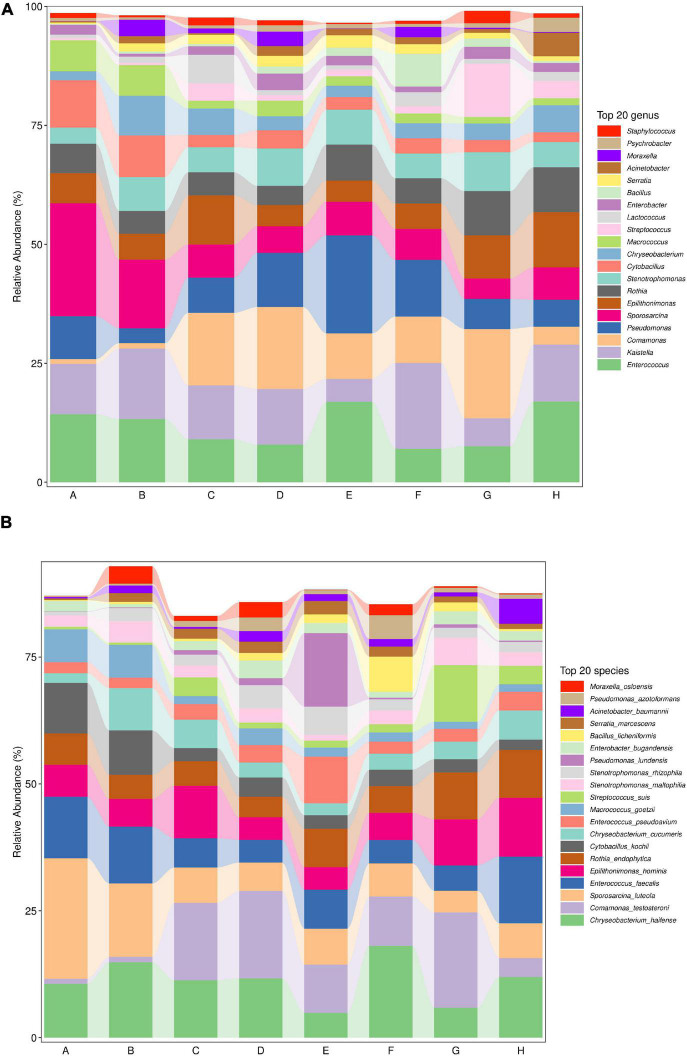
Relative OTU abundance at the **(A)** genus and **(B)** species levels from raw milk samples used for this study. A. Heilongjiang, B. Inner Mongolia, C. Gansu, D. Henan, E. Anhui, F. Jiangsu, G. Chongqing, and H. Hunan.

Milk samples were also used to culture psychrotrophic bacteria that were then identified using 16S rDNA sequencing from pure cultures. We then combined this data with the single-molecule sequencing data to analyze the diversity of psychrotrophic bacteria in raw milk. The most abundant genera in our samples were *Enterococcus*, *Kaistella*, *Commonas*, *Pseudomonas*, *Sporosarcina*, *Epilithonimonas*, *Rothia*, *Stenotrophomonas*, *Cytobacillus*, *Chryseobacterium*, *Macrococcus*, *Streptococcus*, *Lactococcus*, *Enterobacter*, *Bacillus*, *Serratia*, *Acinetobacter*, *Moraxella*, *Psychrobacter*, and *Staphylococcus*. In particular, *Sporosarcina* was the most abundant psychrophilic genus (23.8%) in raw milk in Heilongjiang. *Comamonas* was the most abundant (18.8%) in samples from Hunan and *Pseudomonas* (20.6%) from Anhui. Overall, *Pseudomonas* accounted for 9.0, 3.1, 7.4, 11.4, 20.6, 11.9, 6.3, and 5.7% from Heilongjiang, Inner Mongolia, Gansu, Henan, Jiangsu, Hunan, and Chongqing, respectively (Figure 2A).

The most abundant species were *Chryseobacterium haifense, Comamonas testosteroni, Sporosarcina luteola, Enterococcus faecalis, Epilithonimonas hominis, Rothia endophytica, Cytobacillus kochii, Chryseobacterium cucumeris, Enterococcus pseudoavium, Macrococcus goetzii, Streptococcus suis, Stenotrophomonas maltophilia, Stenotrophomonas rhizophila, Pseudomonas lundensis, Enterobacter bugandensis, Bacillus licheniformis, Serratia marcescens, Acinetobacter baumannii, Pseudomonas azotoformans*, and *Moraxella osloensis* (Figure 2B). *S. luteola* was the most abundant in Heilongjiang (23.8%) and *C. testosteroni* in Henan (17.2%) and *P. lundii* in Anhui (14.5%) raw milk samples (Figure 2B).

### Principal Coordinates Analysis

**FIGURE 3:**
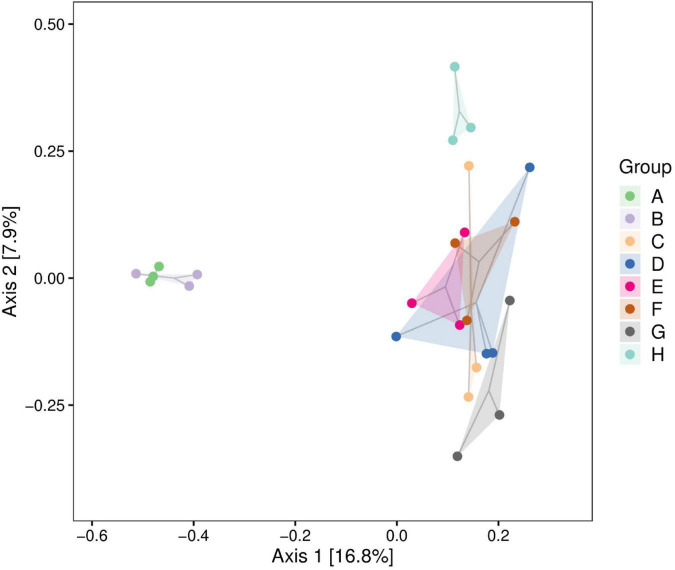
Principal coordinates analysis (PCoA) plot of raw milk samples used in this study. A. Heilongjiang, B. Inner Mongolia, C. Gansu, D. Henan, E. Anhui, F. Jiangsu, G. Chongqing, and H. Hunan.

We analyzed the correlations between community structures of the psychrophilic bacteria in raw milk samples from different regions using PCoA. The Heilongjiang and Inner Mongolia raw milk samples clustered indicating similar communities. Interestingly, these two groups were clearly separated from the remaining sample regions indicating significant differences in community structures (Figure 3).

### Clustering Heat Map Analysis

**FIGURE 4:**
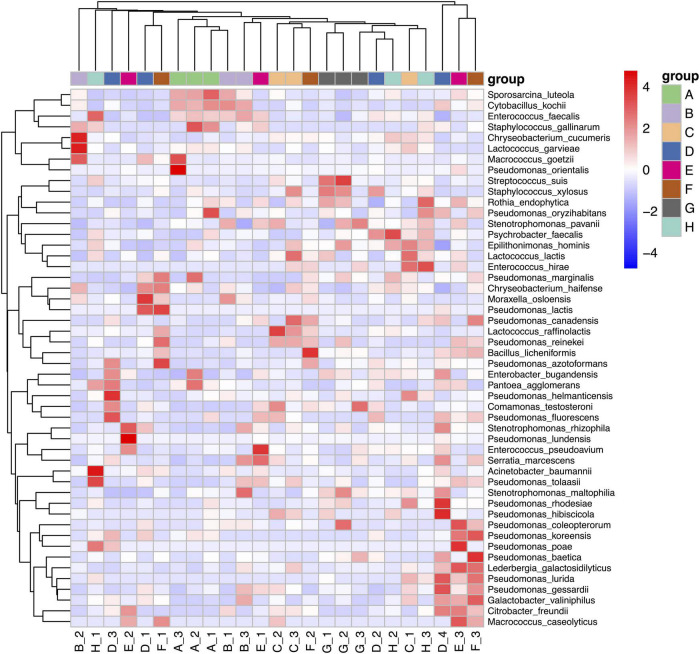
Heatmap analysis of raw milk samples used in this study. A. Heilongjiang, B. Inner Mongolia, C. Gansu, D. Henan, E. Anhui, F. Jiangsu, G. Chongqing, and H. Hunan.

We used clustering heat map analysis to further explore the differences in the community structure of psychrotrophic bacteria using the top 50 relative abundance scores. This analysis utilized the Euclidean distance of the microbial composition data and species were clustered according to Pearson correlation coefficient data. We found that Heilongjiang and Inner Mongolia samples were clustered together and separated from the other groups due to the presence of *S. luteola*, *C. kochii*, and *E. faecalis*. The clustering of Henan, Anhui, and Jiangsu raw milk samples were due to the presence of *P. lurida*, *P. gessardii*, *L. galactosidilyticus*, *G. valiniphilus*, and *C. freundii* (Figure 4).

### Species Difference Analysis of Psychrotrophic Bacteria in Samples

**FIGURE 5:**
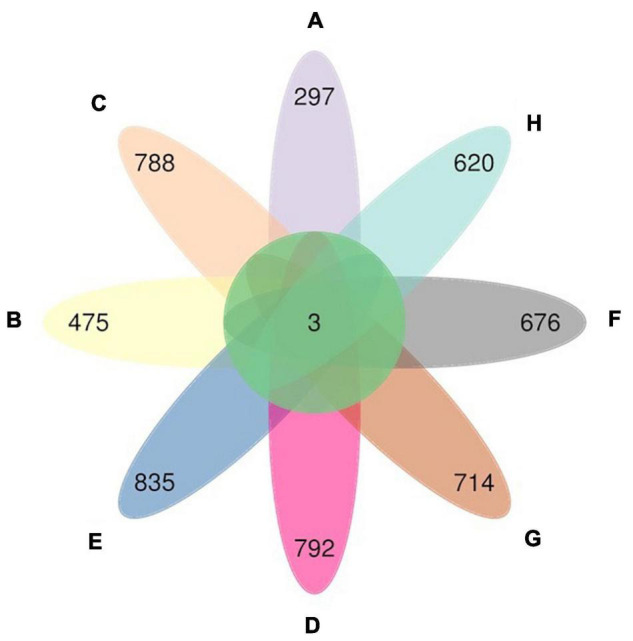
Venny analysis of raw milk samples used in this study. A. Heilongjiang, B. Inner Mongolia, C. Gansu, D. Henan, E. Anhui, F. Jiangsu, G. Chongqing, and H. Hunan.

**FIGURE 6:**
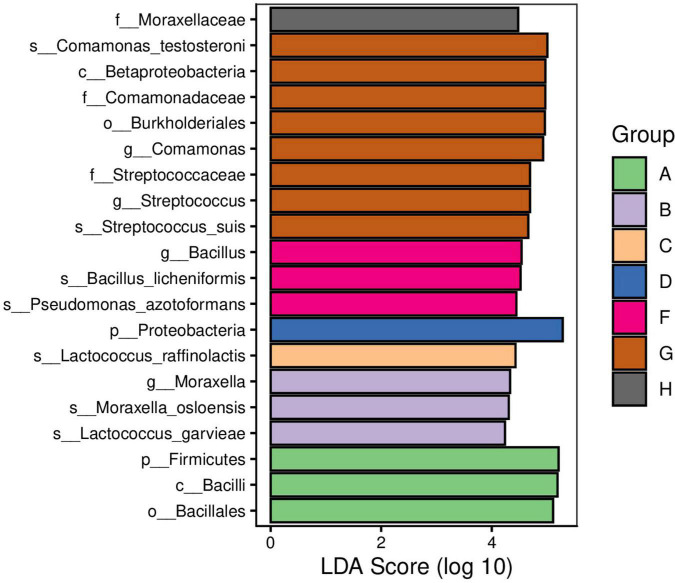
Linear discriminant analysis (LDA) of raw milk samples used in this study. A. Heilongjiang, B. Inner Mongolia, C. Gansu, D. Henan, E. Anhui, F. Jiangsu, G. Chongqing, and H. Hunan.

We used Venny and linear discriminant analyses to categorize the different raw milk bacterial species that we identified from our eight study areas. We found that *E. faecalis* and *R. endophytica* were common species identified in samples from all regions. In addition, the relative abundance of *Bacillus* in Jiangsu samples was significantly greater than for the other regions while *Streptococcus* and *Comamonas* in Hunan samples were significantly greater than other regions (Figures 5, 6).

## Discussion

We isolated 248 psychrophilic bacterial colonies using traditional culture methods and found 21 genera and 59 species that were identified by 16 rDNA gene sequencing. At the genus level, *Pseudomonas*, *Stenotrophomonas*, *Enterococcus*, *Chryseobacterium*, and *Serratia* were the present in the highest proportions and *Pseudomonas* accounted for 58.9% of the total. At the species level, *P. azotoformans*, *P. paralactis*, *P. lactis*, *E. faecalis*, and *P. marginalis* were the present in the highest proportions and *P. azotoformans* accounted for to 16.9% of the total species numbers in our samples. These results were consistent with a previous study where *Pseudomonas*, *Lactococcus*, and *Acinetobacter* accounted for 62% of the total (Von Neubeck et al., 2015). Another study found that the *Pseudomonas* was the most prevalent genus (74.79%) in raw milk and was represented by 25 species, the most common of these species were *P. fragi* (10.92%), *P. lundensis* (6.72%), and *P. fluorescens* (6.72%) (Zhang et al., 2020). Additionally, a study utilizing 595 strains of psychrotrophic bacteria from raw milk samples indicated 90% of the samples contained *Pseudomonas* at proportions as high as 25.7% (Yang et al., 2020).

The traditional culture of microorganisms has numerous advantageous over other methods and is an indispensable technical means for the in-depth exploration of microbial resources in raw milk. The composition of psychrotrophic bacterial microbiota was investigated by submitting the psychrotrophic bacteria database to the raw milk microbiota database. We used single-molecule real-time sequencing to also identify un-culturable microorganisms to understand the total diversity of microorganisms in raw milk. These combined data indicated that *Enterococcus, Kaistella, Comamonas, Pseudomonas, Sporosarcina, Epilithonimonas, Rothia, Stenotrophomonas, Cytobacillus*, *Chryseobacterium*, *Macrococcus*, *Streptococcus*, *Lactococcus*, *Enterobacter*, *Bacillus*, *Serratia*, *Acinetobacter, Moraxella*, *Psychrobacter*, and *Staphylococcus* were the 20 most abundant genus in all samples. This was consistent with the results of previous studies that found *Pseudomonas*, *Acinetobacter*, *Lactococcus*, *Corynebacterium*, and *Streptococcus* as the most abundant genus in raw milk (McHugh et al., 2020). An additional study indicated that *Lactococcus*, *Bacillus*, *Streptococcus*, *Sporosarcina*, *Pseudomonas*, and *Acinetobacter* were the primary psychrophilic bacteria at the genus level in raw milk (Hou et al., 2015). Raw milk samples analyzed in another study indicated and *Pseudomonas*, *Lactococcus*, and *Acinetobacter* were the most common genera (Li et al., 2018). In our study we found that *Enterococcus* posed a high risk for contamination and a previous study of 1,584 batches of raw milk samples from four dairy factories found that *Enterococcus* was presence at levels ranging from 40.6 to 79.7% (Yoon and Lee, 2021). This agreed with another study where the relative abundance of *Enterococcus* in raw yak milk reached levels as high as 36% (Qazalbash et al., 2021).

At the species level we found that the most abundant organisms were *C. haifense, C. testosterone, S. luteola, E. faecalis, E. hominis, R. endophytica, C. kochii, C. cucumeris, E. pseudoavium, M. goetzii, S. suis, S. maltophilia, S. rhizophila, P. lundensis, E, bugandensis, B. licheniformis, S. marcescens, A. baumannii, P. azotoformans*, and *M. osloensis*. A previous study indicated that *E. faecalis*, *S. rhizophila*, *P. lundensis*, and *M. osloensi* were the primary psychrophilic bacteria at the species level in raw milk (Yang et al., 2020). The most common source for *A. baumannii* is environmental pollution and this bacterium can cause pneumonia, meningitis, and wound infections (Engür et al., 2014). We found that the relative abundance of *A. baumannii* in Chongqing was relatively high and this was consistent with previous studies implicating raw milk and commercial infant formula milk powder as sources (Tamang et al., 2014). *Streptococcus* originates primarily in the farm environment and is a common cause of dairy cow mastitis and most animals have no obvious clinical symptoms at the time of infection (Moroni et al., 2006). *S. suis* has been also identified in raw milk in cows with mastitis (Ma et al., 2021). We found *S. suis* in Hunan samples and this may be related to farm management practices.

China is a vast country with many farms, we also analyzed the influence of geographic regions factors on the community structure of psychrotrophic bacteria in raw milk, and the results showed that geographic regions factors significantly influenced the community structure of psychrotrophic bacteria in raw milk, with Heilongjiang and Inner Mongolia clustered together in both principal coordinates analysis and cluster heat map analysis due to their location in the north of China, while Henan, Anhui, and Jiangsu clustered together due to their proximity. This is consistent with previous findings that geographic factors were significant factors influencing the milk microbiota (Guo et al., 2021), while studies on psychrotrophic bacteria had also shown that geographic factors were determining factors attributing to significant influence in the raw milk psychrotrophic bacteria (Yap et al., 2021). In addition, differences in the community structure of psychrotrophic bacteria in raw milk between different geographic regions may be influenced by storage temperature (Vithanage et al., 2016; Hahne et al., 2019; Quinto et al., 2020). The low-temperature storage resulted in an increasing relative abundance of psychrotrophic bacteria (Yang et al., 2020), the psychotropic bacterial community structure diversity in the summer was higher than that in the winter (Vithanage et al., 2016). Cold storage tests using raw water buffalo milk indicated that the microbial community structure changed with storage time. The first 24 h were dominated by *Lactococcus* and *Streptococcus* while at 72 h *Pseudomonas* and *Acinetobacter* dominated and latter achieved levels more than 60% (Li et al., 2016). A comparison of the effects of different cold storage conditions on the microorganism content of raw milk indicated that *Pseudomonas* isolates also dominated all temperatures and were more than 80% at 2 and 4°C (Vithanage et al., 2017), however, only 8–10% at 10 and 30°C (Hahne et al., 2019), however, another study found that all *Pseudomonas* can grow at 2–25°C (Meng et al., 2019), 67% *Pseudomonas* can grow at 40°C (Caldera et al., 2016). Differences were observed between days in Henan, Anhui, and Jiangsu samples that may be related to farm management or disinfection procedures. Disinfection of farm milking equipment and acid or alkaline disinfection are usually performed every other day.

## Conclusion

This is the first study to analyze the diversity of psychrophilic bacteria in raw milk in different regions of China by a combination of traditional culture and PacBio single-molecule real-time sequencing. The community structure of psychrotrophic bacteria in raw milk was very different across regions. In this study, we found that the community structure and diversity of psychrotrophic bacteria in raw milk in Heilongjiang and Inner Mongolia were similar although they differed significantly from the other study regions. Additionally, differences were observed between days in Henan, Anhui, and Jiangsu samples. These procedures assisted in a more accurate understanding of the community structure of psychrophilic bacteria in raw milk from different regions and can be used to develop timely individual control strategies on a case or a reginal basis in order to ensure milk safety.

## Data Availability Statement

The datasets presented in this study can be found in online repositories. The names of the repository/repositories and accession number(s) can be found below: https://bigd.big.ac.cn, CRA005869.

## Author Contributions

BD: conceptualization, methodology, investigation, and writing – original draft. LM: methodology. HL: data curation. NZ: conceptualization and supervision. YZ and SZ: writing – review, supervision, and funding acquisition. JW: conceptualization and funding acquisition. All authors contributed to the article and approved the submitted version.

## Conflict of Interest

The authors declare that the research was conducted in the absence of any commercial or financial relationships that could be construed as a potential conflict of interest.

## Publisher’s Note

All claims expressed in this article are solely those of the authors and do not necessarily represent those of their affiliated organizations, or those of the publisher, the editors and the reviewers. Any product that may be evaluated in this article, or claim that may be made by its manufacturer, is not guaranteed or endorsed by the publisher.

## Footnotes

1
https://imagej.nih.gov/ij/download.html
